# Proteomic approach to discover human cancer viruses from formalin-fixed tissues

**DOI:** 10.1172/jci.insight.143003

**Published:** 2020-11-19

**Authors:** Tuna Toptan, Pamela S. Cantrell, Xuemei Zeng, Yang Liu, Mai Sun, Nathan A. Yates, Yuan Chang, Patrick S. Moore

**Affiliations:** 1 Hillman Cancer Center, University of Pittsburgh, Pittsburgh, Pennsylvania, USA.; 2 Institute of Medical Virology, University Hospital Frankfurt, Goethe University, Frankfurt am Main, Germany.; 3 Biomedical Mass Spectrometry Center and; 4 Department of Cell Biology, University of Pittsburgh, Pittsburgh, Pennsylvania, USA

**Keywords:** Virology, Cancer, Proteomics

## Abstract

The challenge of discovering a completely new human tumor virus of unknown phylogeny or sequence depends on detecting viral molecules and differentiating them from host molecules in the virus-associated neoplasm. We developed differential peptide subtraction (DPS) using differential mass spectrometry (dMS) followed by targeted analysis to facilitate this discovery. We validated this approach by analyzing Merkel cell carcinoma (MCC), an aggressive human neoplasm, in which ~80% of cases are caused by the human Merkel cell polyomavirus (MCV). Approximately 20% of MCC have a high mutational burden and are negative for MCV, but are microscopically indistinguishable from virus positive cases. Using 23 (12 MCV^+^, 11 MCV^–^) formalin-fixed MCC, DPS identified both viral and human biomarkers (MCV large T antigen, CDKN2AIP, SERPINB5, and TRIM29) that discriminate MCV^+^ and MCV^–^ MCC. Statistical analysis of 498,131 dMS features not matching the human proteome by DPS revealed 562 (0.11%) to be upregulated in virus-infected samples. Remarkably, 4 (20%) of the top 20 candidate MS spectra originated from MCV T oncoprotein peptides and confirmed by reverse translation degenerate oligonucleotide sequencing. DPS is a robust proteomic approach to identify potentially novel viral sequences in infectious tumors when nucleic acid–based methods are not feasible.

## Abstract 2

Differential peptide subtraction (DPS) is an application of differential mass-spectrometry followed by targeted analysis that can facilitate tumor virus and biomarker discovery.

## Graphical abstract

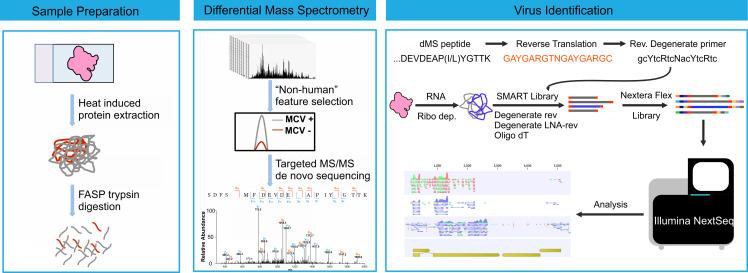

## Introduction

Seven human viruses are responsible for approximately 15% of the tumor burden world-wide. This phylogenetically heterogeneous group of viruses differ extensively in their genome sizes, nucleic acid composition, and replication mechanisms (1). Likewise, the discovery processes for each of these 7 tumor viruses has varied and evolved closely with technological advances, particularly in molecular biology. Epstein-Barr virus (EBV or human herpesvirus [HHV4]; ref. 2), a large double stranded DNA herpesvirus, was first identified in 1964 based on classic microbiology detection practices in cell culture and electron microscopy. Hepatitis B virus (HBV), unculturable at the time, was found by serologic screening in 1965 (3). The discovery of human T-lymphotropic virus-1 (HTLV-1), a retrovirus, was facilitated by reverse transcriptase assays in 1980 (4), and — although many strains of human papillomaviruses (HPV) were already identified by 1983 — cervical cancer–associated HPV strains were only identified through strain-specific DNA Southern hybridization studies (5). Hepatitis C virus (HCV), a flavivirus, was found by cDNA library screening in 1989 (6).

Molecular subtractive techniques have been most recently used to determine the infectious etiologies of Kaposi sarcoma (KS) and Merkel cell carcinoma (MCC). In 1993, fragments of the KS herpesvirus (KSHV/HHV8) genome were cloned using representational difference analysis (RDA), a DNA-based subtractive process that can isolate foreign nucleic acids from the human genome (7, 8). In 2008, Merkel cell polyomavirus (MCV) transcripts were found by digital transcriptome subtraction (DTS), an in silico RNA subtractive process taking advantage of a timely expansion in sequencing capabilities, databases, and search engines (9, 10).

Virus-associated cancers are biological accidents, detrimental to both the host and the viral pathogen (11). The cancer virus is generally not actively replicating (latency or pseudolatency) in cancerous cells, which would otherwise tend to kill the host cell. However, latent viral transcript levels tend to be reduced relative to cellular or lytic viral transcript levels (12). Latent viral proteins, on the other hand, can have exceptional stability (13), be expressed by noncanonical translation (14, 15), and can circumvent cellular protein degradation mechanisms (13). This is thought to be a viral strategy to reduce immunoproteasomal peptide processing to escape host immune responses against latent viral proteins (16). Based on these biologic features of tumor viruses, we pursued a protein-based detection method that may be useful for tumors in which RNA is unavailable or in which viral transcript levels are too low to be routinely detected. A protein-based virus discovery method using cross-reactive antibodies to viral proteins has been described for polyomaviruses (pan–polyomavirus IHC test, P-PIT) (17, 18). Since P-PIT depends on conserved epitopes within a class of known viruses, it cannot identify unique nucleic acid or peptide sequences from a new agent.

We sought an unbiased approach for deep peptide sequencing to differentiate human from foreign peptides belonging to potentially novel viruses that can make use of archival pathology samples. To achieve this, we developed a methodology called differential peptide subtraction (DPS) using label-free differential mass spectrometry (dMS) to quantify relative peptide abundance between complex samples (19–21). The advantages of DPS are that it is able to interrogate protein abundance; can identify novel peptides; makes use of formalin-fixed, paraffin-embedded (FFPE) tissues; and, if no pathogen is found, can reveal unique cellular protein biomarkers that may improve diagnosis and prognosis of a target disease.

## Results

Unbiased DPS was performed on polyomavirus-positive and -negative MCC. MCC is a highly aggressive human skin cancer, 80% of which is etiologically associated with MCV (9). Virus-positive MCCs express viral small T (sT) and large T (LT) antigen oncoproteins and have a low mutation burden (22). In this subset of MCCs, the MCV genome clonally integrates into the host chromosome and acquires mutations or deletions, resulting in the translation of C-terminally truncated LT proteins, which vary in size from tumor to tumor. In contrast, virus-negative MCC, although microscopically indistinguishable from virus-positive MCC, carry high mutational burdens and driver somatic mutations that phenocopy MCV infection (23). We used MCV^–^ (*n* = 11) and MCV^+^ (*n* = 12) MCC FFPE tissues, and we processed them in a blinded fashion to determine whether DPS can distinguish the presence of a tumor virus de novo in human tissues without prior knowledge of the virus identity or sequence.

**Figure 1:**
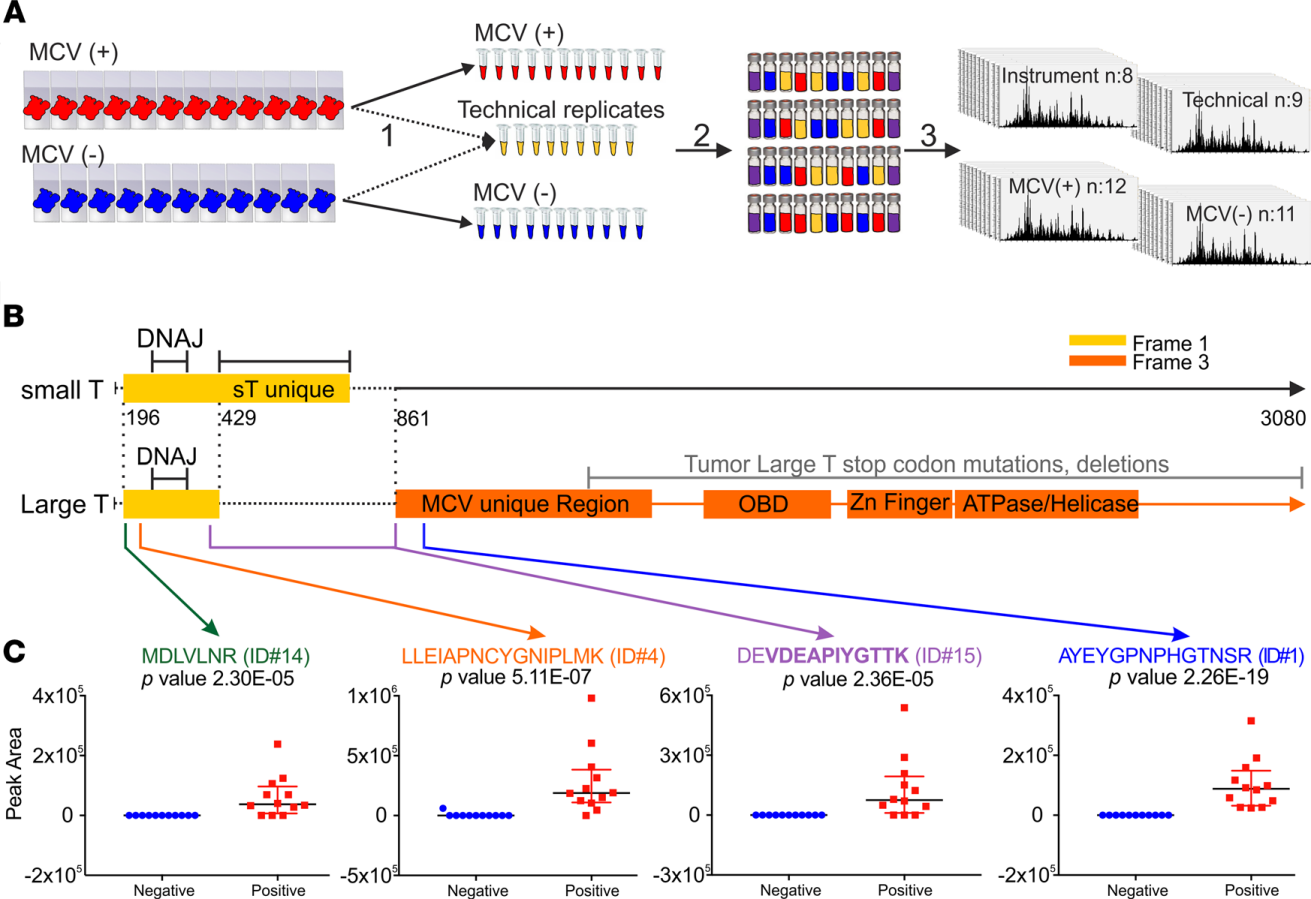
DPS can detect de novo the presence of a tumor virus. (**A**) Workflow for dMS sample processing and instrumental analysis. Step 1: deparaffinization, antigen retrieval, and lysis. A total of 10 μL from each sample (*n* = 23) was combined and aliquoted into 9 technical replicates. Step 2: FASP digestion. Each sample was normalized to 30 μg. A total of 750 fmol of ovalbumin was added as an internal standard. A pooled instrument control was made by combining 5 μL from each sample (*n* = 32). Samples (*n* = 33) were reordered. Step 3: nLC-MS/MS analysis. Injection of ~0.2 μg on to C18 Picochip column Orbitrap Velos Pro and analysis. (**B**) Schematic illustration of MCV T antigen transcripts. Small T (yellow, Frame 1) and Large T (yellow, Frame 1; orange, Frame 3) transcripts from the early region including start, splice, and termination sites are shown. Both small T and large T encode DnaJ domain. Small T and MCV unique domains, origin binding (OBD), zinc finger, ATPase, and helicase domains are depicted. The location of mutations and deletions found in MCC tumor large T are highlighted with a gray line. Positions of the 4 MCV peptides identified by dMS analysis are indicated with green, orange, purple, and blue arrows. (**C**) Dot plots for the relative abundance of identified viral peptides in MCV^+^ (red, *n* = 12) versus negative (blue, *n* = 11) MCC samples**.** Peptides and their rankings (Table 1) are shown in green (ID#14), orange (ID#4), purple (ID#15), and blue (ID#1). Data are shown as mean ± SD. *P* values were based on 2-sided equal variance Student’s *t* test.

Polyomavirus status was initially determined by MCV LT antigen immunohistochemical staining (Supplemental Table 1; supplemental material available online with this article; https://doi.org/10.1172/jci.insight.143003DS1). Proteins were extracted from FFPE tissues and digested using filter-aided sample preparation–based (FASP-based) tryptic digestion and analyzed by nano-flow liquid chromatography tandem MS (nLC-MS/MS) (Figure 1A). High-resolution full-scan (MS1) mass spectra and low-resolution tandem (MS2) mass spectra were recorded on a hybrid ORBItrap Velos mass spectrometer (Figure 1A). Four types of experimental samples were included in the experimental design: 11 MCV^–^ samples, 12 MCV^+^ samples, 9 sample processing replicates, and 1 instrument control sample (Figure 1A and Supplemental Figure 1). A data set of 498,131 high-resolution MS1 features (Supplemental Table 2) was extracted from the raw mass spectral data using the MaxQuant (v1.6.0.1) proteomic software package (24, 25). Subsequently, all MS1 features that could be identified by searching the MS2 spectra against a human Uniprot protein database (downloaded in February 2013 with 87,662 entries) were removed. The log_2_-transformed intensities of the unidentified proteomic features were analyzed with a 2-tailed Student’s *t* test to select features that exhibit significant differences in relative abundance between MCV^+^ and MCV^–^ tumor samples. Filtering for spectral features with *P* < 0.01 and at least a 10-fold higher intensity in MCV^+^ samples compared with MCV^–^ samples returned 562 features. Targeted nLC-MS/MS analysis was used to collect MS2 spectra for the 20 most significant features ranked by ascending *P* value. Manual de novo sequencing identified aa sequence tags greater than 5 aa long for 11 of 20 selected features (Table 1). A Blast search against UniProtKB revealed that 4 of these peptides matched to the MCV T antigen protein sequence (Table 1 and Figure 1B). MCV sT and LT antigens are derived from differentially spliced transcripts and share a 78-aa N-terminus, nucleotide 196–429 (Frame 1). LT splices after the first exon into a C-terminal exon (738 aa, nucleotide 861–3080; Frame 3), whereas the sT transcript reads through this splice donor site at nucleotide position 429 to generate a protein having an identical N-terminal domain with LT but a different C-terminal domain (Figure 1B). The localization of 4 peptides identified by targeted nLC-MS/MS analysis are shown in Figure 1B. The 2 peptides on the left (green; Table 1 identification no. 14 [ID#14]; orange, ID#4) are common to sT and LT, whereas the other 2 (purple, ID#15; blue, ID#1) correspond to LT. There is a partial match to sT (aspartate-glutamate, DE) for the third peptide (purple, ID#15), which spans the splice junction between exon 1 and 2 of LT. The relative abundance of the identified MCV peptides in the infected versus control samples (Figure 1C) show that a human tumor virus in tumor tissues can generate sufficient protein to be identified de novo from tumor tissue. Thus, the comparison of proteomic profiles from infected and control tissues allows identification of new proteins without a priori knowledge of the protein sequence.

**Figure 2:**
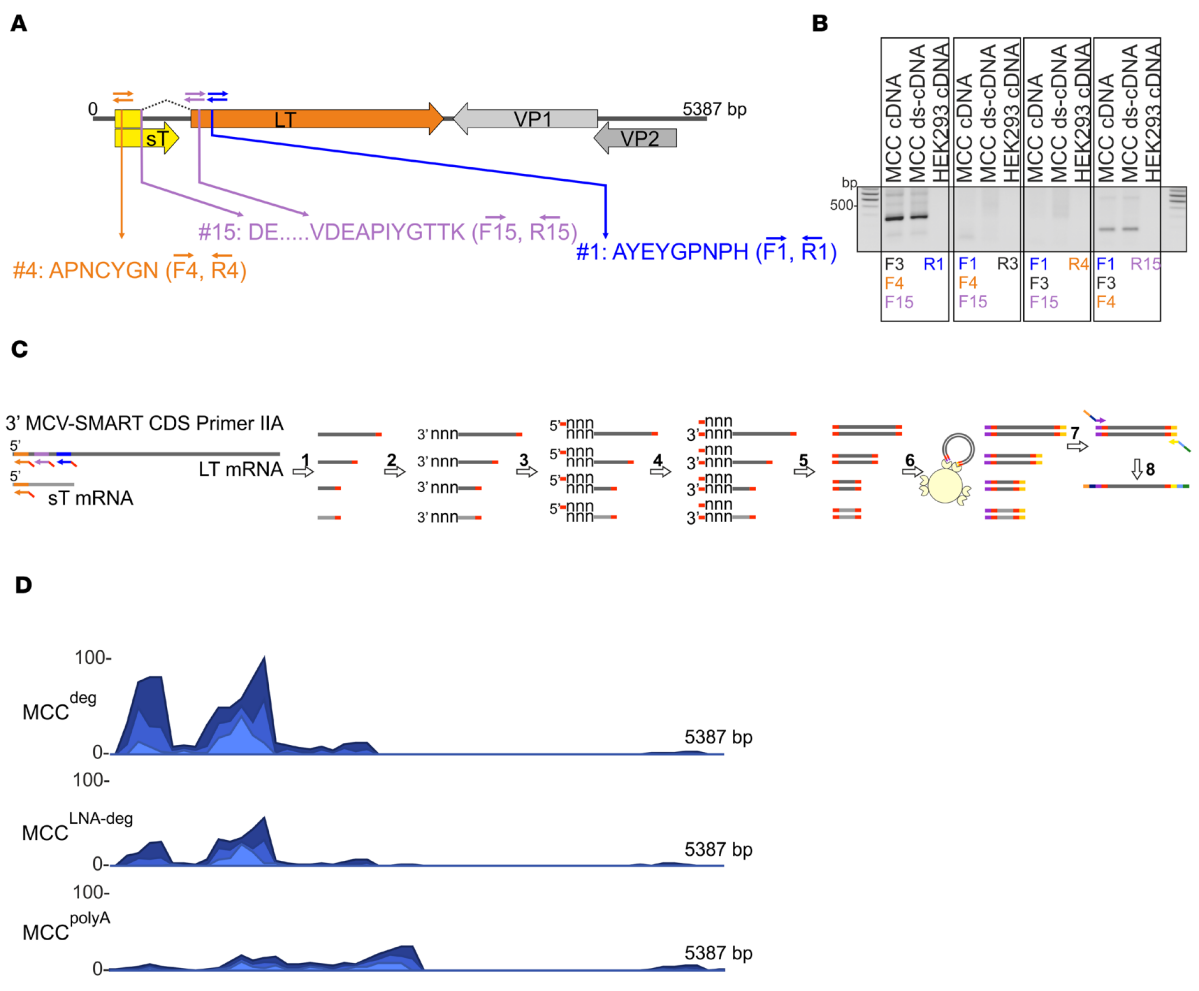
dMS-identified peptides facilitate identification of viral sequences by NGS with cDNA libraries generated using degenerate oligonucleotides. (**A**) Schematic illustration of the MCV genome. Early (LT, yellow and orange; sT, yellow) and late (VP1, light gray; VP2, dark gray) region open reading frames are shown. The corresponding positions of the 3 MCV peptides identified by dMS (features 4, 15, and 1) and degenerate primer binding sites are shown in orange, purple, and blue arrows, respectively. (**B**) RNA extracted from MCC tissue (tissue R16–67) or HEK293 cells were subjected to cDNA synthesis with random hexamers and, additionally, second-strand synthesis for the MCC sample (double-stranded cDNA; ds-cDNA). cDNAs were amplified using the indicated combinations of degenerate primers (Supplemental Table 3) corresponding to the peptide sites highlighted in light blue (F1, R1), violet (F15, R15) and orange (F4, R4). F, forward; R, reverse. F3 and R3 (black) are non-MCV primers. (**C**) Library generation using SMART oligonucleotides and Nextera DNA Flex. Step 1: 3′ SMART CDS Primer IIA (Supplemental Table 4) mediated first-strand synthesis. Step 2: Tailing by RT. In the cDNA reaction, nontemplated bases (nnn) are added to the ends of nascent cDNA by the terminal transferase activity of RT. Step 3: SMARTer IIA oligo anneals to nontemplated bases at cDNA ends (nnn). Step 4: Template switch and extension at 3′ end. The RT polymerase switches strands to transcribe the complement of the oligonucleotide, leaving the SMART adaptor at both ends of cDNA. Step 5: Long-distance PCR with single 5′ PCR Primer IIA amplifies libraries. Step 6: Bead-linked transposomes mediate the simultaneous fragmentation of ds-cDNA and the addition of Illumina sequencing primers using Nextera DNA Flex. Step 7: Reduced-cycle PCR amplification amplifies sequencing-ready DNA fragments and adds indexes and adapters. Step 8: Sequencing-ready fragments are washed and pooled. (**D**) NGS coverage maps of MCC RNA-seq libraries. RNA-seq reads were obtained from 3 different samples to compare the efficiency of MCV read recovery using various primer pool sets for cDNA and library generation (Supplemental Table 3). Ribo-depteted MCC RNA (R11–65) was subjected to cDNA synthesis with SMART-degenerate oligo pool (MCC^deg^), LNA modified SMART-degenerate oligo pool-SMART (MCC^LNA-deg^), and modified oligo-dT-SMART (MCC^polyA^) and then subjected to library generation using Nextera DNA Flex application. Standardized coverage depths (reads) for comparison purposes are indicated on the *y* axis for each alignment.

In the case of a novel virus, dMS-identified peptides will not have a match in the databases; nevertheless, this information can facilitate the recovery of the viral genome sequence. To this end, we sought to trace the nonhuman dMS–identified peptides back to their genetic origins by next-generation sequencing (NGS) with cDNA libraries generated using degenerate oligonucleotides based on the identified peptide sequences (Supplemental Table 3). In designing degenerate primers (DP), we aimed to avoid primer sequences with 6-fold codon nucleotide variants (L, S, and R) and to maximize the number of 2-fold codon variants (D, E, Y, N, K), thereby maintaining moderate binding-specificity while reducing oligo degeneracy. In line with this, peptide areas with X residues (Table 1) representing either an L or an I (3-fold degeneracy sites) codon, which are indistinguishable by MS, were excluded. In addition to nonhuman matches (ID#1, ID#4, and ID#15; Table 1), we included peptide ID#3, which was only a partial match to human in a Blast search. Based on the in silico reverse translation, forward and reverse primers were designed for a total of 4 peptides (Supplemental Table 3). First, we tested the binding efficiency of these DP by a low-cycle reverse transcription PCR (RT-PCR) (Figure 2A). Four different sets of combinations of forward and reverse DP, and cDNA template from a MCV^+^ MCC sample, were used for the PCR reaction (Figure 2A). Combinations of forward (F) 4, reverse (R) 1 and F4, R15 primers resulted in 400 and 200 bp PCR products, respectively, which were confirmed to be derived from MCV by sequencing (Figure 2B; see complete unedited blots in the supplemental material).

For the library generation to perform NGS analysis, degenerate oligonucleotides were fused to Switching Mechanism at 5′ End of RNA Template (SMART) adaptor sequence (SMART-deg, 25 nt) (Supplemental Table 4) and used for cDNA synthesis, as described previously (26) (Figure 2C). To demonstrate the principle and efficiency of this procedure, mixtures of SMART-degenerate oligonucleotides or a modified oligo(dT) SMART primer were used to facilitate RT from viral or viral and host RNA, respectively. Due to high degeneracy of these primers, we sought to increase their specificity and designed another set of primers by addition of a number of locked-nucleic acid (LNA) modifications for indicated bases (Supplemental Table 4). Using degenerate (deg), LNA modified degenerate (LNA-deg) primer pools, and modified oligodT (polyA), we generated MCC^deg^, MCC^LNA-deg^, and MCC^polyA^ SMART cDNAs, respectively, which were then processed into 3 Nextera DNA Flex libraries and subsequently sequenced using NextSeq 500 (Figure 2D). Fifty-eight million to 68 million reads per sample were obtained, which were processed and mapped to a combined reference index from GRCh38 and MCV (JF813003) annotations (Table 2). Normalization procedures to account for different sequencing depths among the 3 libraries include conversion of data to transcripts-per-million (TPM) read-outs and trimmed mean of M values (TMM). We detected 7.3 and 2.6 times more MCV reads in degenerate oligo primed RNA-seq samples compared with polyA-based sequencing reads. In addition, reads from MCC^deg^ and MCC^LNA-deg^ largely mapped upstream of the DP binding sites within the T antigen region. Hence, this strategy can facilitate the identification of a viral genome sequences even in cases where the dMS peptides do not match to previously identified pathogens.

The label-free dMS method not only identified differentially expressed viral peptides within a complex mixture, but also proteins that can serve as prognostic biomarkers. A total of 17,921 unique human peptides from 2832 corresponding protein groups were quantified, and the peptide intensity values were log_2_ transformed (Supplemental Table 5). A 2-tailed Student’s *t* test was used for statistical comparison between MCV^+^ and MCV^–^ peptide intensity values. Significant proteins were selected if more than half of the identified peptides from a protein were significant (*P* < 0.05), and single peptide identifications were excluded from the analysis. A total of 38 proteins showed significantly increased abundance, whereas 8 proteins were decreased in abundance in MCV^+^ samples. The list of identified peptides for these proteins are included in Supplemental Table 6.

**Figure 3:**
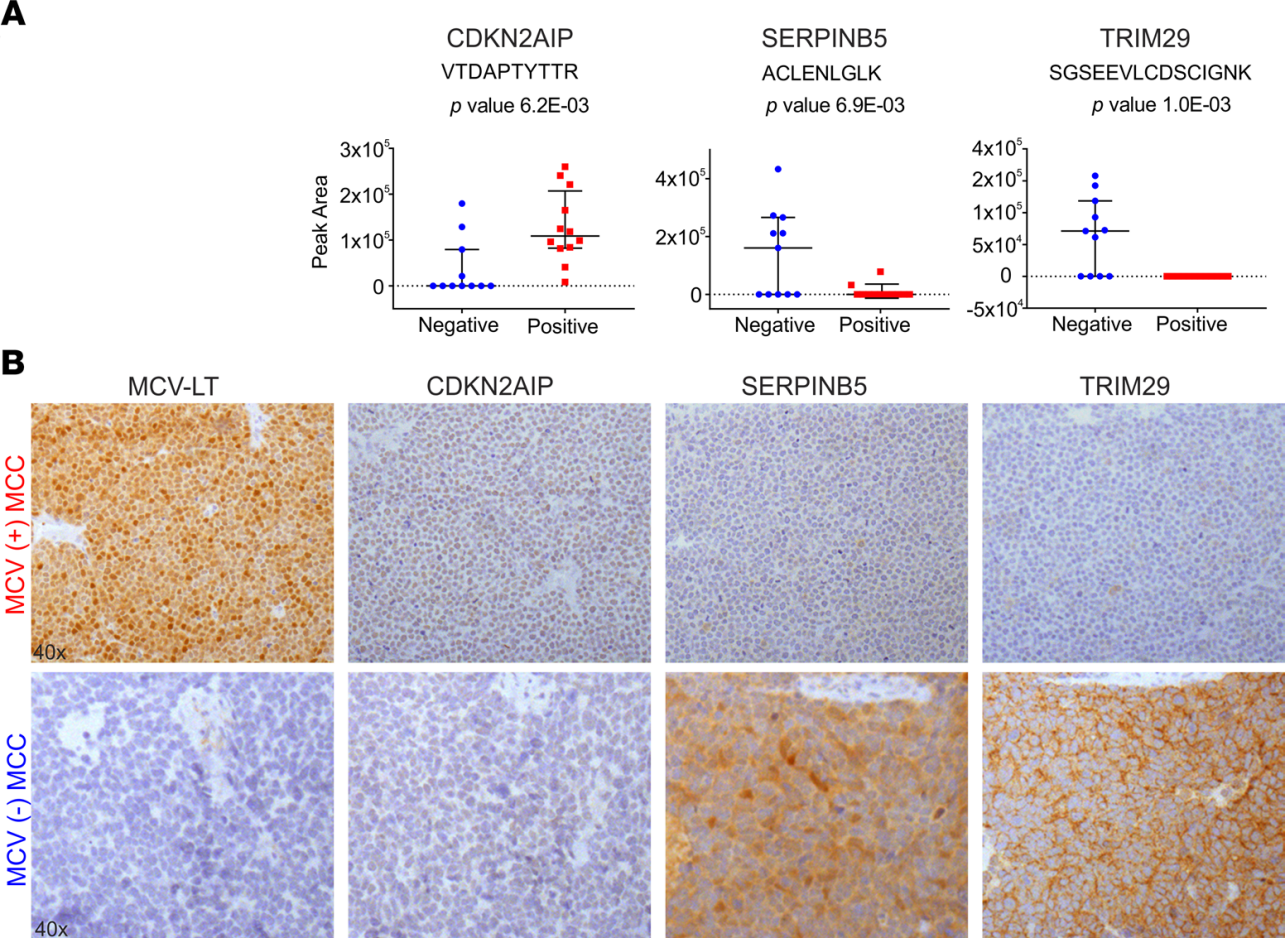
DPS can identify differentially expressed human peptides as potential biomarkers. (**A**) Dot plots for the relative abundance of identified human peptides in MCV^+^ (red, *n* = 12) versus negative (blue, *n* = 11) MCC tumor samples. Data are shown as mean ± SD. *P* values were based on 2-sided equal variance Student’s *t* test. (**B**) IHC staining of MCC TMA. R10–115 and R15–03 are representative MCV^+^ (upper panel) and MCV^–^ (lower panel) MCC cases, respectively. According to the IHC staining results, we detected SERPINB5 and TRIM29 in MCV^–^ cases and in none of the MCV^+^ cases, as predicted by dMS analysis. MCV LT expression was detected using CM2B4 is a monoclonal antibody. Original magnification, 40×.

To validate differentially expressed human peptides as potential biomarkers, 5 MCV^+^ and 4 MCV^–^ MCC tissue cores, together with control tissues, were used to generate a tissue microarray and were analyzed for the expression of CDKN2AIP, SERPINB5, and TRIM29 by IHC (Figure 3). Consistent with dMS results, we found loss of TRIM29 and SERPINB5 expression and higher levels of CDKN2AIP expression in all MCV^+^ MCC cases (Figure 3A). These results suggest a role of MCV T antigens in the regulation of SERPINB5 and TRIM29 expression (Figure 3B).

## Discussion

In this study, we provide a nLC-MS/MS–based protocol to compare tissues and identify differentially expressed peptides and potential prognostic markers. This is a peptide/proteome subtraction process that is analogous to the mRNA/digital transcriptome subtraction (DTS) originally used to discover MCV (9). Importantly, the high DPS de novo identification rate for MCV peptides in the context of the entire human tumor tissue proteome shows that this approach is promising. We anticipate that it can supplement RNA-based analyses of suspected infectious cancers, especially for tumors in which it is difficult to obtain sufficient RNA for sequencing.

The top 20 unsupervised candidate MS feature sequences (after differential and database subtraction that were present in MCV^+^ but not MCV^–^ samples) were manually determined. These 20 peptide sequences were then aligned to the human proteome using the basic local alignment search tool (BLASTP), which revealed 4 of these 20 peptides to be of MCV origin. These 4 peptides map to the N-terminus of the MCV T antigen oncoprotein complex, including peptides common to sT and LT, and to the beginning of the second exon in LT, which are common to the coding regions of the truncated LT proteins found in all the MCV^+^ MCC tumors (Figure 1B).

Modern virus discovery only requires a discovery of a single unique nucleotide sequence to recover the entire viral sequence by gene walking. We show that, starting from 3 unique peptides, NGS of degenerate cDNA from the MCV^+^ MCC tumor library recovers unique viral nucleic acid sequences that can allow full viral characterization. Although this approach proved to be more efficient than poly-A NGS, LNA modifications to the oligonucleotides used for cDNA generation did not seem to improve the outcome. We anticipate that sequentially performing these steps (first DPS on formalin-fixed tumor tissues followed by degenerate NGS of candidate peptide coding sequences using well-accessioned tumor tissue RNA libraries) is a viable strategy to find and characterize human tumor viruses, particularly in rare tumors.

DPS relies on comparison of a viral cancer proteome to a matched control nonviral tumor proteome. Other known paired viral/nonviral tumors that could be similarly tested include head-and-neck carcinoma, nasopharyngeal carcinoma, Burkitt lymphoma, and hepatocellular carcinoma (1). In our study, spectral features with a *P* < 0.01 and at least a 10-fold–higher intensity in MCV^+^ tumors returned 562 features. Targeted nLC-MS/MS analysis was used to collect MS2 spectra for the 20 most significant features ranked by ascending *P* value, which enabled the identification of candidate viral peptides. Effective ranking and prioritization is important because de novo sequencing remains a largely tedious and slow manual process. The entire protocol from the processing of blinded specimens to the unbiased identification of the viral protein consumes less than 3 weeks of laboratory time.

For some of the cancer types, however, a well-defined control group might not be available. In such cases, statistical analysis for hierarchical clustering of the samples might be useful. To specifically address this potential problem, we used unsupervised hierarchical clustering to investigate the possibility of using proteomic profiles to accurately classify MCV samples into 2 groups, viral-positive and viral-negative groups. The best classification result was obtained using the proteomic profiles for proteins associated with virus-related biological processes. Nineteen of 22 samples were correctly classified (86% accuracy; Supplemental Figure 2). MCV features remained significantly different between the 2 cluster groups, despite the drop in their significance ranking, supporting the potential of applying unsupervised clustering for classification of samples with unknown viral status.

An alternative approach in the absence of matched negative control tissues is the generation of a reference database comprising human MS/MS peptide features. This reference database could then be used for DPS in silico subtraction of universal “human” peptides from tumor MS/MS profiles, leaving candidate “nonhuman” peptide sequences. Such a MS/MS database (the proteome equivalent of the nucleotide RefSeq database) does not currently exist. Such a database would also be highly dependent on machine and sample characteristics, as well as biological characteristics (e.g., single nucleotide polymorphisms, posttranscriptional modifications, and posttranslational modifications) that would make universal comparisons difficult. Following subtraction, degenerate NGS using the candidate nonhuman MS/MS spectra to design oligonucleotides could be used to search for viral sequences. This strategy would not only circumvent the need for well-matched histological tissue controls, but it would also reduce the cost, time, and manual labor needed in evaluation steps. As with nucleotide DTS (10), an in silico DPS analysis may miss a viral pathogen if commensal or endogenous virus peptide features are mistakenly assigned as “human” in the comparison database.

Even when no new virus is found, DPS has utility for identifying human protein biomarkers. We identified 38 human proteins significantly increased and 8 proteins decreased in MCV^+^ versus MCV^–^ MCC samples, including SERPINB5, a reported tumor suppressor also known as mammary serine protease inhibitor (MASPIN) (27), and TRIM29, a ubiquitin E3 ligase that may act as a scaffold protein in the DNA damage response (28). Loss of TRIM29 expression promotes invasion of skin squamous cell carcinoma cells by altering distribution of keratins (29). Loss of expression of these 2 proteins might contribute to a more aggressive disease course for MCV^–^ compared with MCV^+^ MCC; however, larger-cohort studies are needed to confirm these initial findings. These and other differentially expressed proteins can be readily examined as potential prognostic biomarkers for MCV^+^ and MCV^–^ MCC tumors or as biomarkers to differentiate MCV^–^ MCC from other small round cell neuroectodermal cancers.

DPS also offers advantages over RNA-seq–only searches for cases where latency-associated viral transcripts are significantly less abundant than cellular transcripts. At present, DPS is more time consuming than NGS and requires tissue-matched negative-control samples. Thus, it should be seen an extension rather than a replacement for RNA-seq analysis in virus discovery. DPS, however, has a critical advantage in making use of archival tumor FFPE tissues in which RNA is degraded. Development of a platform-independent human MS/MS reference database may markedly expand the potential for uncovering new human pathogens using DPS.

## Methods

### Cell line, tissues, tissue microarray generation, and IHC.

HEK293 cells (ATCC) were maintained DMEM (10-013, Cellgro) supplemented with 10% FBS (MilliporeSigma).

MCV^+^ and MCV^–^ MCC tumors were obtained from Cooperative Human Tissue Network (CHTN). Based on the MCV LT expression levels determined by CM2B4 staining, 11 MCV^–^ and 12 MCV^+^ tumors were selected for the dMS study. Among those cores from 5 MCV^+^, 4 MCV^–^ tumor FFPE blocks and a series of normal tissues (spleen, colon, brain, prostate, skin, adrenal gland, kidney, lung, uterus, and tonsil) were used to generate a tissue microarray at UPMC Hillman Cancer Center Tissue and Research Pathology services.

Slides were deparaffinized in xylene and rehydrated in a series of ethanol solutions. Endogenous peroxidase activity was blocked by incubation of the slides with 3% hydrogen peroxide for 15 minutes. Epitope retrieval was performed using 1 mM EDTA buffer pH 8.0 at 125°C for 3 minutes and 90°C for 15 seconds in an antigen retrieval chamber (Decloaking chamber, Biocare medica). After blocking (protein block, serum free, Dako), monoclonal antibody CM2B4 generated by standard methods of immunizing mice with KLH-derivatized SRSRKPSSNASRGA peptide from the MCV T antigen (22) (0.6 μg/mL mAb, 1:1500), and commercial antibodies CDKN2AIP (1:400, sc-81841, Santa Cruz Biotechnology Inc.), MASPIN/SERPINB5 (1:400, sc-271694, Santa Cruz Biotechnology Inc.), and ATDC/TRIM29 (1:400, sc-376125, Santa Cruz Biotechnology Inc.) were diluted in (1% BSA, 0.1% gelatin, 0.5% Triton-X, 0.05% sodium azide in PBS, pH 7.4), were applied to each section overnight at 4°C in a humidified chamber. Following extensive rinsing steps in TBS, sections were incubated with mouse Envision Polymer (Dako) for 30 minutes at room temperature, reacted with deaminobenzidine (DAB, Dako), and counterstained with hematoxylin (Dako). Images were acquired using Olympus microscope AX70 (Olympus Co.). All other chemicals were purchased from MilliporeSigma.

### Sample selection, preparation for dMS.

A total of 23 FFPE MCC tissue samples were selected on the basis of immunohistochemical staining that determined the presence/absence of MCV. The samples were anonymized to assure that analysts were blind to the MCV status of the tissues until the proteomic sample preparation and MS analysis were complete. Samples were sectioned to a 10 μm thickness using a microtome and stored on standard microscope slides.

### Preparation of FFPE tissue for MS.

Deparaffinization was achieved with 2 xylene washes (3 minutes each), rehydrated with serial ethanol washes (100%, 100%, 95%, and 70% for 1 minute each), and washed with LC-MS grade water twice for 3 minutes each. After deparaffinization, 100 μL lysis buffer (300 mM Tris [pH 8.0], 100 mM DTT, 2% SDS) was added to each tissue sample, followed by 30 minutes of sonication, 1 hour of incubation at 95°C, and 2 hours of incubation at 65°C. After centrifugation at 17,000*g* for 10 minutes at room temperature, the supernatants containing the extracted proteins were transferred to new eppendorf tubes, and the Pierce 660 nm Protein Assay kit with the IDCR packet (Thermo Fisher Scientific) was used to determine the total protein content.

Sample aliquots containing 30 μg of total protein were digested with trypsin using the Filter Aided Sample Preparation (FASP) protocol (30). In brief, the protein samples were added to YM30 Microcon microcentrifuge filters (MilliporeSigma) and washed 3 times with 200 μL of urea buffer (100 mM Tris-HCl [pH 8.0], 8M urea), each with 15 minutes centrifugation at 14,000*g* at room temperature. Alkylation was performed by incubating at room temperatures for 20 minutes in 100 μL of urea buffer with 20 mM iodoacetamide. Samples were then washed 3 times with 100 μL urea buffer and then 3 times with 100 μL 50 mM ammonium bicarbonate, each with 10 minutes centrifugation at 14,000*g* at room temperature. A total of 1.2 μg Sequencing Grade TPCK-treated trypsin (Promega) was then added to each sample for overnight digestion in a humidified 37°C incubator. The resultant peptides were desalted using C18 Supelco cartridges (Supelco), SpeedVac dried, and then reconstituted in 30 μL 0.1% formic acid for analysis. All other chemicals were purchased from MilliporeSigma.

Quality control samples were used to evaluate variability introduced by proteomic sample processing and MS analysis. A set of 9 sample processing controls were created by combining equal amounts of undigested protein from the 23 extracted FFPE samples and processed alongside the experimental samples. A pool FFPE protein extract was divided into 9 aliquots and processed together with the experimental samples to access sample preparation performance. A pooled instrument control sample was generated by combining equal volumes of all the digested samples and analyzed multiple times to monitor the stability of the MS system over time. All sample identities were blinded to eliminate analyst bias and processed using a balanced block design to reduce variability introduced during sample processing and nLC-MS/MS analysis. The mean coefficient of variation (CV) for all quantified human peptides was used to characterize the biological (CV, ~90%) and technical (CV, ~30%) variation in the individual and replicate samples, respectively (Supplemental Figure 1).

### MS and data processing.

Complex mixtures of proteolytic peptides (0.2 μg for each injection) were analyzed by nLC-MS/MS with a nano Acquity UHPLC (Waters Corporation) interfaced to a hybrid Orbitrap Velos Pro mass spectrometer (Thermo Fisher Scientific). Peptide separation was carried out on a C18 PicoChip 25 cm column (New Objective) with a 66-minute linear gradient of 2%–35% solvent B (acetonitrile/0.1% formic acid) at a 300 nL/min flow rate. The mass spectrometer was operated in positive ionization mode with an electrospray voltage of 1.9 kV and capillary temperature of 275°C. Ion sampling and accumulation was controlled with automatic gain control (AGC) and maximum injection time settings of 1,000,000 and 500 ms for full-scan high-resolution (MS1) mass spectra, and 5000 and 100 ms for the low-resolution ion trap tandem (MS2) mass spectra, respectively. Data-dependent acquisition recorded a full-scan MS1 spectrum at a resolution setting of 60,000 followed by 13 MS2 spectra at normalized collision energy setting of 35 with dynamic exclusion enabled. Separate nLC-MS/MS analyses that collect MS2 spectra on predefined precursor ions were performed using an isolation width of 2 *m/z* units and a relative collision energy setting of 35.

The raw mass spectrometry data were analyzed with MaxQuant software version 1.6.0.1 (24) that incorporates the Andromeda (25) protein identification search engine and label-free quantification tools. MS2 spectra were searched against the UniProt human proteome database (February 2013 release; uniprot.org) using standard ORBItrap parameters and a reversed decoy database strategy that limits false peptide identifications rates to 1% or less. Briefly, a precursor mass tolerance setting of 20 and 4.5 ppm were used for the first and main database search, respectively. A mass tolerance setting of 0.5 Da was used for the MS2 fragment ions. Search enzyme specificity was defined as trypsin with maximum of 2 missed cleavages, fixed Cysteine carbamidomethylation, and variable methionine oxidation and protein N-terminal acetylation modifications. A minimum peptide length setting of 7 was used, and the maximum number of modification per peptide was limited to 5. The “match between runs” and “matched unidentified” settings were enabled to prompt quantification of high-resolution MS1 features, regardless of the peptide sequence identification status.

Raw MS data files, together with MaxQuant quantification results, have been deposited to the ProteomeXchange consortium via the MassIVE partner repository (data set identifier PXD021520, http://proteomecentral.proteomexchange.org/cgi/GetDataset?ID=PXD021520).

### RNA extraction and SMART-library generation.

Total RNA was isolated from MCV^+^ MCC tumor (R1165) using TRIzol (Ambion Inc.) and was treated with TURBO DNase (Thermo Fisher Scientific). RNA quality was examined by 2100 Bioanalyzer (Agilent Technologies) before (RNA integrity number [RIN] value 5.3) and after ribosomal RNA depletion using RiboMinus Eukaryote kit (Thermo Fisher Scientific) according to the manufacturer’s recommendations. Ribosome depleted samples were subsequently used for MCC-SMART library preparation. Libraries were prepared using the SMARTer PCR cDNA synthesis kit (Clontech) according to the manufacturer’s recommendations with the following modifications: SMART fusion primers were designed, which have the SMART sequence (5′-AAGCAGTGGTATCAACGCAGAGTAC-3′) added to the 5′ end of each dMS-identified MCV- or human-specific degenerate reverse primer listed in Supplemental Table 3. dMS-SMART-DP mix or a modified oligo(dT) primer (3′ SMART CDS primer IIA) were used to prime first-strand cDNA synthesis. Reaction mixtures consisting of 3.5 μL of RNA (~300 ng), 1 μL of 24 μM SMART primer mix (1.2 μM final concentration for each), or 1 μL of 12 μM 3′ SMART CDS primer IIA (5′-AAGCAGTGGTATCAACGCAGAGTACT_(30)_N_–1_N-3′, where N = A,C,G, or T and N_–1_ = A,G, or C) were heated at 72°C for 3 minutes, and then the temperature was lowered to 47°C (0.1°C/min slope) for 2 minutes before the addition of 5.5 μL of master mix (2 μL of 5× first-strand buffer, 0.25 μL of 100 mM DTT, 1 μL of 10 mM dNTP mixture, 1 μL of 12 μM SMARTer IIA oligonucleotide, 0.25 μL of 40 U/μL RNase inhibitor, and 1 μL of 100 U/μL SMARTScribe RT) (Clontech). cDNA synthesis reaction mixtures of clinical specimens were incubated at 47°C for a total of 90 minutes, terminated at 70°C for 10 minutes, and brought to 4°C before the addition 0.1 μL RNase H (5 U/μL, New England Biolabs). Reaction mixtures were incubated at 37°C for 20 minutes, subsequently kept at 4°C, and adjusted to 50 μL with water.

SMART cDNA was amplified by long-distance PCR on a thermocycler as follows using Advantage II reagents (Clontech): 7.5 μL SMART cDNA, 7.5 μL 10× Advantage 2 PCR buffer, 1.5 μL 50× dNTP mix (10 mM), 1.5 μL 5′ PCR primer IIA (12 μM), 1.5 μL 50× Advantage 2 polymerase mix, and 55.5 μL water (total of 75 μL). Reaction mixtures were cycled as follows: 95°C for 1 minute; 35 cycles of 95°C for 15 seconds, 65°C for 30 seconds, and 68°C for 3 minutes, followed by a hold at 4°C.

Amplified SMART cDNA was purified with AMP-Pure magnetic beads (Beckman Coulter Genomics) using a ratio of 1.8× beads to sample according to the manufacturer’s recommendations. Libraries were eluted in 30 μL of 10 mM Tris-Cl (pH 7.5) and then quantified on Agilent 2100 Bioanalyzer (Agilent) reagents.

### NGS library generation, sequencing, and analysis.

Nextera DNA Flex kit was used to generate libraries from SMART-cDNA templates following the manufacturer’s instructions, and sequencing was carried out on a NextSeq500 platform (Illumina Inc.) for 2 × 75 paired-end reads. Fastq files were imported into CLC Genomics Workbench 20.0 software (QIAGEN), paired-end reads 1 and 2 were merged, and duplicate reads were removed. Reads were filtered for *Q* scores above 30 and trimmed for quality (limit, 0.05) and ambiguity (2-nt maximum), and the Illumina and SMART adaptor sequence were removed. Reads below 20 nt were discarded, and paired-end reads were aligned to combined reference index from GRCh38 (hg38) and MCV (JF813003) or to individual reference genomes. The following alignment settings were applied: mismatch, 2; insertion, 3; deletion, 3; length fraction, 0.8; and similarity fraction, 0.8. Sequencing data are deposited at NCBI GEO Platform accession number GSE157610 (https://www.ncbi.nlm.nih.gov/geo/query/acc.cgi?acc=GSE157610).

### Statistics.

Feature selection was based on a combination of statistical significance and fold change difference. A 2-tailed equal variance Student’s *t* test on the log_2_-transformed intensities was used to determine the significance of difference between MCV^+^ and MCV^–^ samples for all high-resolution MS1 features that consist of an “isotope group” without a corresponding human peptide sequence identification. Zero peptide intensity values were imputed with one-tenth of the global minimum of nonzero values to enable log_2_ transformation and fold-change calculation. Unidentified MS1 features with at least a 10-fold increase in MCV^+^ samples were ranked in an ascending order according to the Student’s *t* test *P* values. Twenty unidentified MS1 features with the highest significance were subject to targeted nLC-MS/MS analysis. The targeted MS2 spectra were interpreted by manual de novo sequence analysis (31) (Supplemental Figure 3–6) and the identified sequence was confirmed with synthesized peptide standards. A representative select ion chromatogram depicts the relative abundance of Feature 1 peptide AYEYGPNPH(158)NSR in individual MCV^+^ and MCV^–^ patient samples (Supplemental Figure 7).

### Study approval.

Tissues were obtained from CHTN and examined under the University of Pittsburgh IRB 86-22: UPCI Tissue Banking Protocol.

## Author contributions

TT, YC, PSM, and NAY designed the experiments. TT, PSC, YL, and XZ performed the experiments. TT performed RNA-seq, IHC, PCR, and data analysis. PSC, MS, and XZ performed dMS and related data analyses. NAY, YC, and PSM supervised the project. TT, PSC, XZ, NAY, YC, and PSM wrote the paper. YC and PSM contributed equally to this work.

## Supplementary Material

supplemental data

supplemental Table 2

supplemental Table 5

supplemental Table 6

## Figures and Tables

**Table 2:**
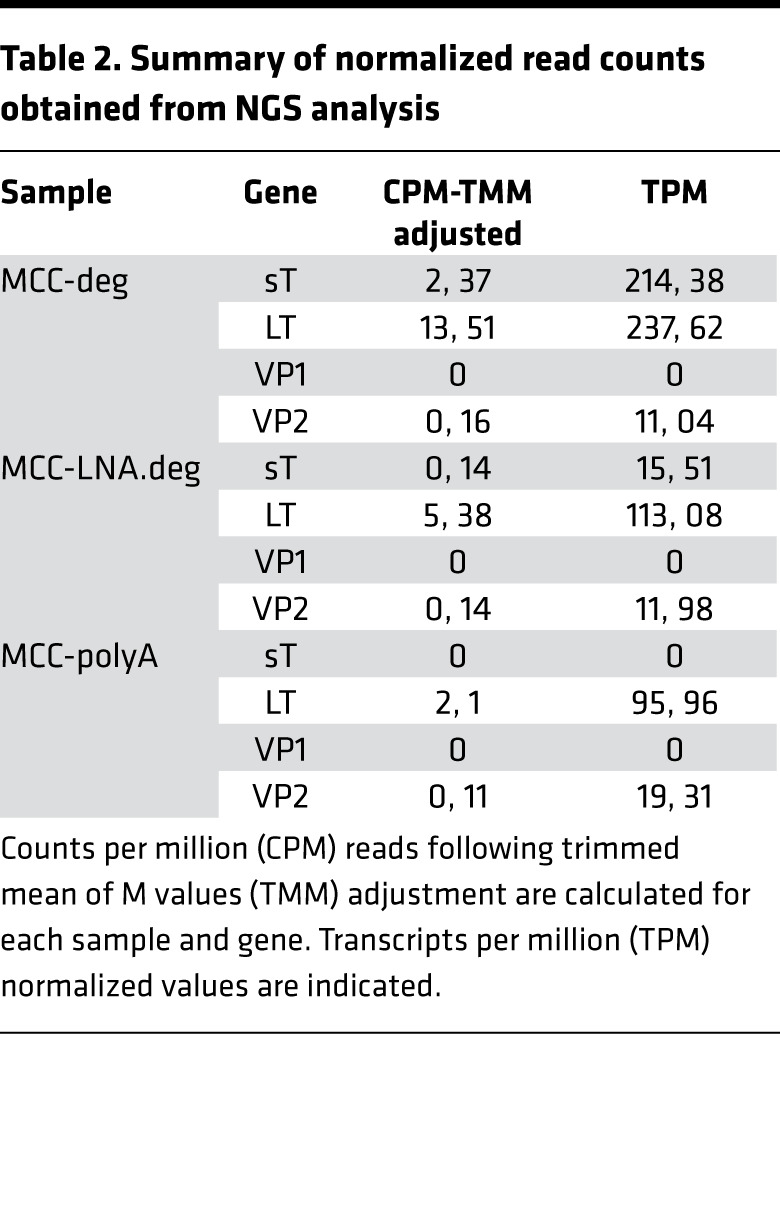
Summary of normalized read counts obtained from NGS analysis

**Table 1:**
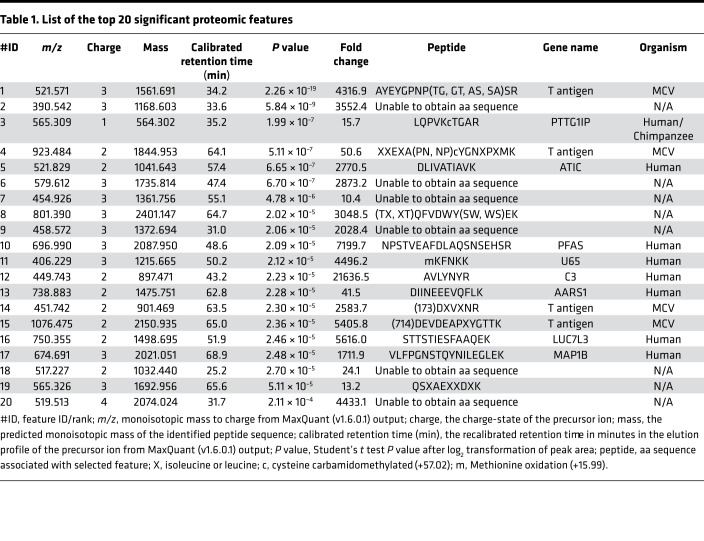
List of the top 20 significant proteomic features

## Version 1 10/15/2020

In-Press Preview

## Version 2 11/19/2020

Electronic publication
