# Supplementary material for: Proteomic approach to discover human cancer viruses from formalin-fixed tissues
Source: JCI Insight. 2020 Nov 19;5(22):e143003. doi: 10.1172/jci.insight.143003 (PMC7710300; doi:10.1172/jci.insight.143003)
Supplement: supplemental data [file jciinsight-5-143003-s064.pdf]

Proteomic approach to discover human cancer viruses from formalin-fixed tissues

Tuna Toptan[1,2], Pamela S. Cantrell[3], Xuemei Zeng[3], Yang Liu[3], Mai Sun[3], Nathan A. Yates[3,4*], Yuan Chang[1,*], Patrick S. Moore[1,*]

Supplemental Tables and Figures

Supplemental Table 1. Immunohistochemistry staining results of MCC cases used for this study.

MCC Cases	CM2B4
R10-115	+++
R10-121	+++
R10-164	-
R11-43	-
R11-65	+++
R12-31	+++
R14-02	+++
R14-05	-
R14-07	-
R14-33	-
R14-34	-
R15-03	-
R15-11	+++
R15-12	-
R15-13	+++
R15-22	+++
R15-23	+++
R16-03	-
R16-41	+++
R16-42	-
R16-67	+++
R16-68	-
R16-69	+++

Supplemental Table 2: List of high-resolution MS1 features without a corresponding human peptide sequence identification.

Supplemental Table 3. Degenerate and LNA-modified degenerate primers used for PCR analysis in **Figure 2**

#ID	Peptide	BlastP	Reverse translation	Forward DP	Reverse DP	LNA-Reverse DP
1	**AYEYGPNPH**(..)**NSR**	MCV	GCNTAYGARTAYGGNCCNAAYCCNCAY	gaRtaYggNccNaaYcc	ggRttNggNccRtaYtc	g+gRt+tN+ggNccRtaYtc
3	**LQPVKcTGAR**	Human/ Chimpanzee	YTNCARCCNGTNAARTGYACNGGNGCNMGN	ccNgtNaaRtgYacNgg	ccNgtRcaYttNacNgg	c+cN+gt+RcaYttNacNgg
4	XXE**XAPNCYGNXPXMK**	MCV	GCNCCNAAYTGYTAYGGNAAY	gcNccNaaYtgYtaYgg	ccRtaRcaRttNggNgc	cc+Rt+aR+caRttNggNgc
15	(..)**DEVDEAP(I/L)YGTTK**	MCV	GAYGARGTNGAYGARGCNCCN	gaYgaRgtNgaYgaRgc	gcYtcRtcNacYtcRtc	g+cY+tc+RtcNacYtcRtc

DP: degenerate primer. Peptide identification numbers (#ID) from **Table 1** are given in the first column.

Supplemental Table 4. SMART-Degenerate and LNA-modified degenerate primers used for NGS library generation.

#ID	Rerverse DP	SMART Reverse DP	LNA-Reverse DP	SMART-LNA-Reverse DP
1	ggRttNggNccRtaYtc	aagcagtggtatcaacgcagagtacggRttNggNccRtaYtc	g+gRt+tN+ggNccRtaYtc	aagcagtggtatcaacgcagagtacg+gRt+tN+ggNccRtaYtc
3	ccNgtRcaYttNacNgg	aagcagtggtatcaacgcagagtacccNgtRcaYttNacNgg	c+cN+gt+RcaYttNacNgg	aagcagtggtatcaacgcagagtacc+cN+gt+RcaYttNacNgg
4	ccRtaRcaRttNggNgc	aagcagtggtatcaacgcagagtacccRtaRcaRttNggNgc	cc+Rt+aR+caRttNggNgc	aagcagtggtatcaacgcagagtaccc+Rt+aR+caRttNggNgc
15	gcYtcRtcNacYtcRtc	aagcagtggtatcaacgcagagtacgcYtcRtcNacYtcRtc	g+cY+tc+RtcNacYtcRtc	aagcagtggtatcaacgcagagtacg+cY+tc+RtcNacYtcRtc

DP: degenerate primer. Peptide identification numbers (#ID) from Table 1 are given in the first column. Positions for the LNA base modifications are indicated with (+). LNA : Locked nucleic acid (LNA). SMART primer sequence is highlighted in blue.

Supplemental Table 5. List of identified human peptides and associated quantification information.

Supplemental Table 6. List of peptides from proteins with significant difference between MCV (+) and control samples.

Supplemental Figure 1

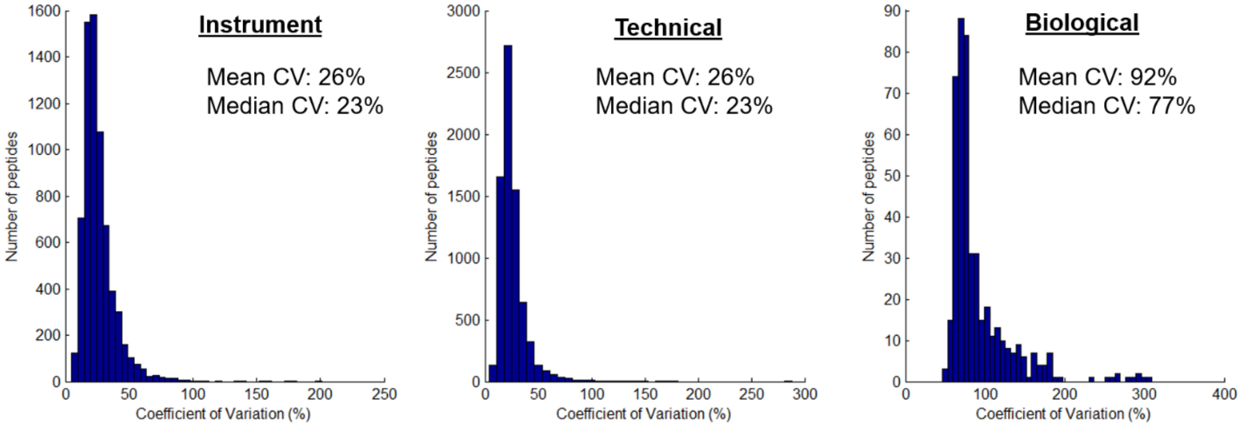

Histogram distribution of the coefficient of variation (CV%) for instrument replicates, sample preparation replicates (technical) and biological samples. For each sample type, only peptides with no missing values were included in the calculation.

Supplemental Figure 2

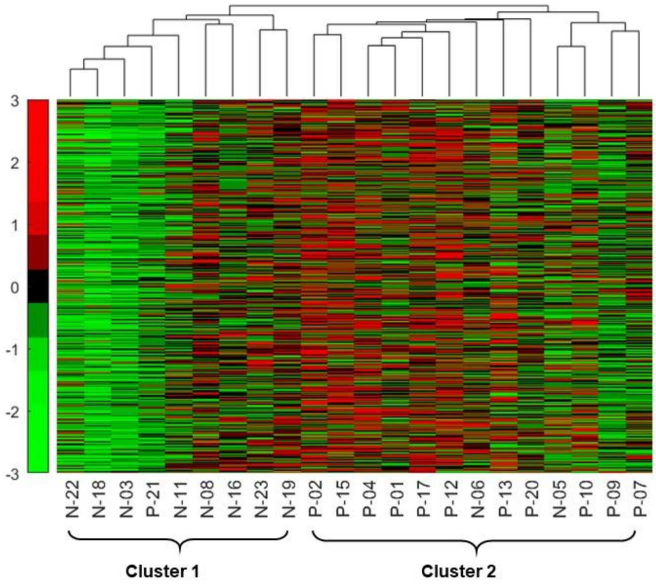

Hierarchical clustering of samples based on the intensity values of peptides from viral related proteins. A total of 235 identified proteins are involved in viral related biological processes including viral transcription, viral process, viral translation termination and re-initiation, viral mRNA export from host cell nucleus, viral release from host cell, viral entry into host cells and virion assembly according to David Bioinformatics Resources 6.8, NIAID/NIH. Sample distance was based on Spearman’s correlation, and the linkage was based on average linkage. The intensity values were standardized for each peptide with Z-transformation. Green and red colors in the clustering represent under- and over-expression, respectively. Sample #14 was excluded to its low overall intensities for all identified peptides. Peptides with more than 50% missing values were also excluded from clustering analysis. N: MCV negative, P: MCV positive samples.

Supplemental Figure 3

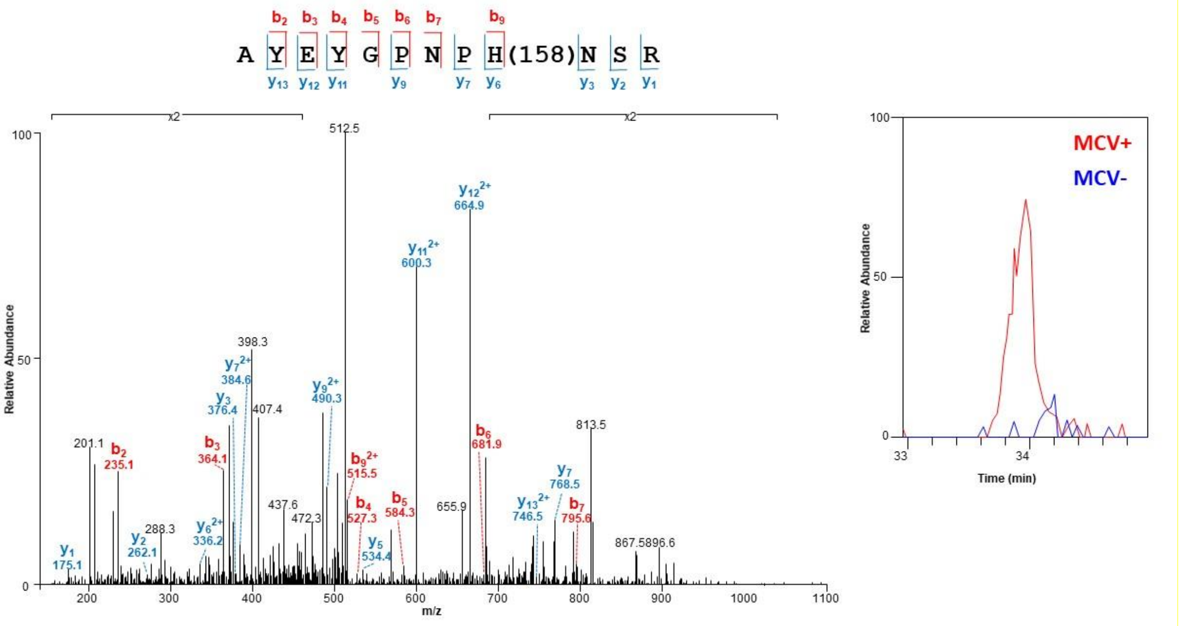

Tandem mass spectrum of proteomic feature #1 from Table 1. Spectrum was acquired by targeted collision activated dissociation (CAD). The tryptic peptide ion m/z 521.6 (p-value 2.26E-19 and fold change 4316.9) has a manual de novo amino acid sequence tag AYEYGPNPH(158)NSR. The amino acid sequence gap at 158 could be GT, TG, AS, or SA. Representative MCV positive and negative shown as MS selected ion chromatogram.

Supplemental Figure 4

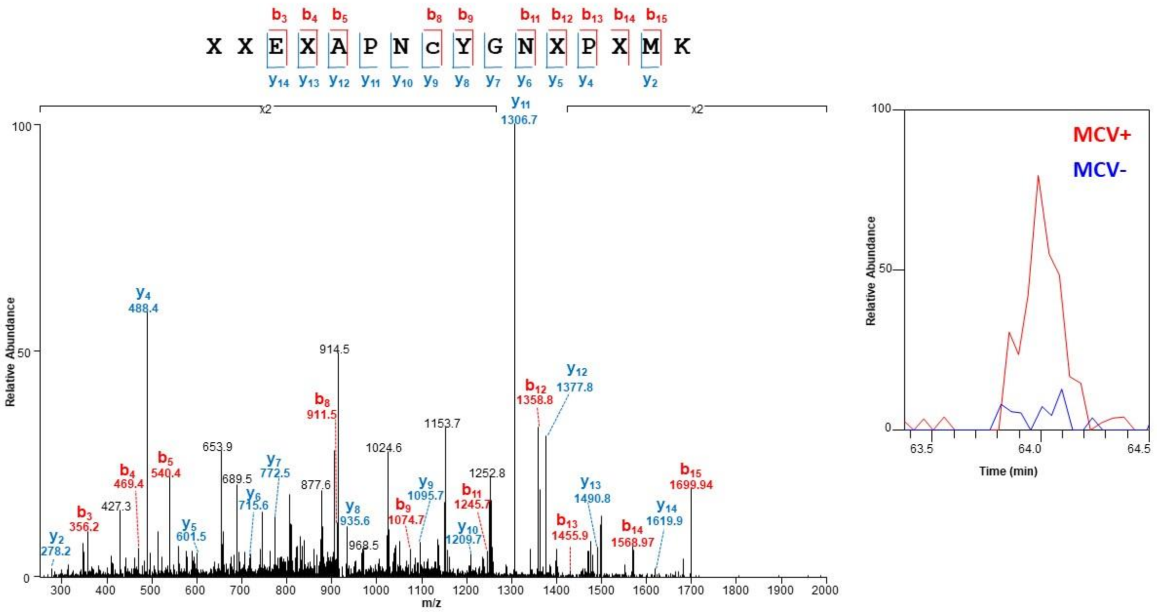

Tandem mass spectrum of proteomic feature #4 from Table 1. Spectrum was acquired by targeted collision activated dissociation (CAD). The tryptic peptide ion *m/z* 923.5 (p-value 5.11E-07 and fold change 50.6) has a manual *de novo* amino acid sequence tag XXE**XA**PNc**YGNXPXMK**. The amino acids “X” refer to isoleucine or leucine and “c” is a cysteine residue with fixed modification carbamidomethylation (+57.02). The first two amino acids could have been XX, EP, or EP; however, EP and PE amino acids were excluded on the basis of mass accuracy. Representative MCV positive and negative shown as MS selected ion chromatogram.

Supplemental Figure 5

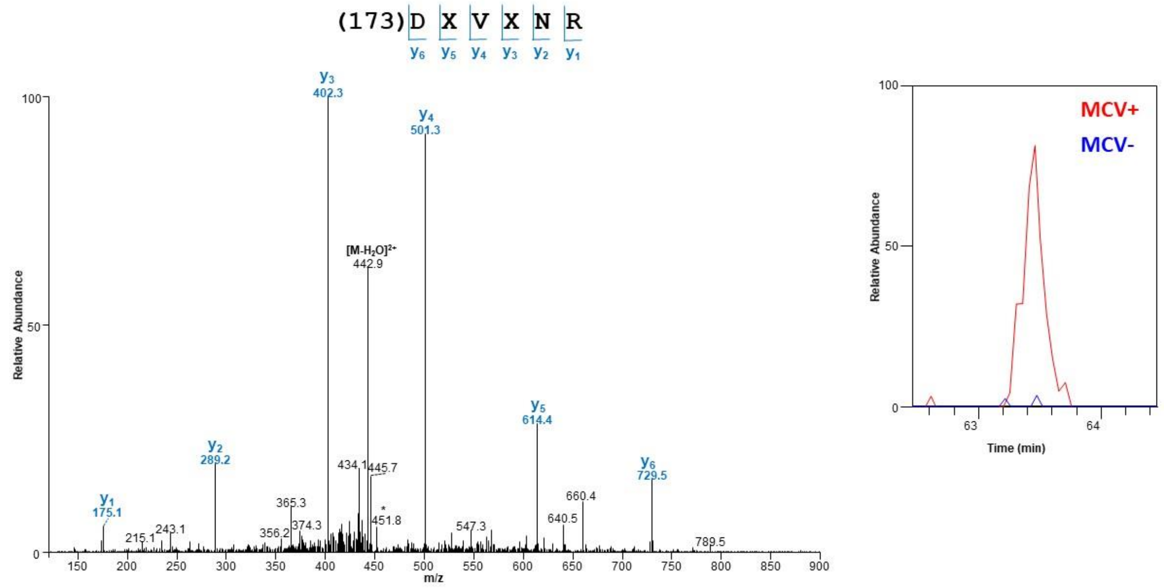

Tandem mass spectrum of proteomic feature #14 from Table 1. Spectrum was acquired by targeted collision activated dissociation (CAD). The tryptic peptide ion *m/z* 451.7 (p-value 2.30E-05 and fold change 2583.7) has a manual *de novo* amino acid sequence tag (173)**DXVXNR**. The amino acids “X” refer to isoleucine or leucine. Not enough information from the spectrum could determine the N-terminal amino acids. Representative MCV positive and negative shown as MS selected ion chromatogram.

Supplemental Figure 6

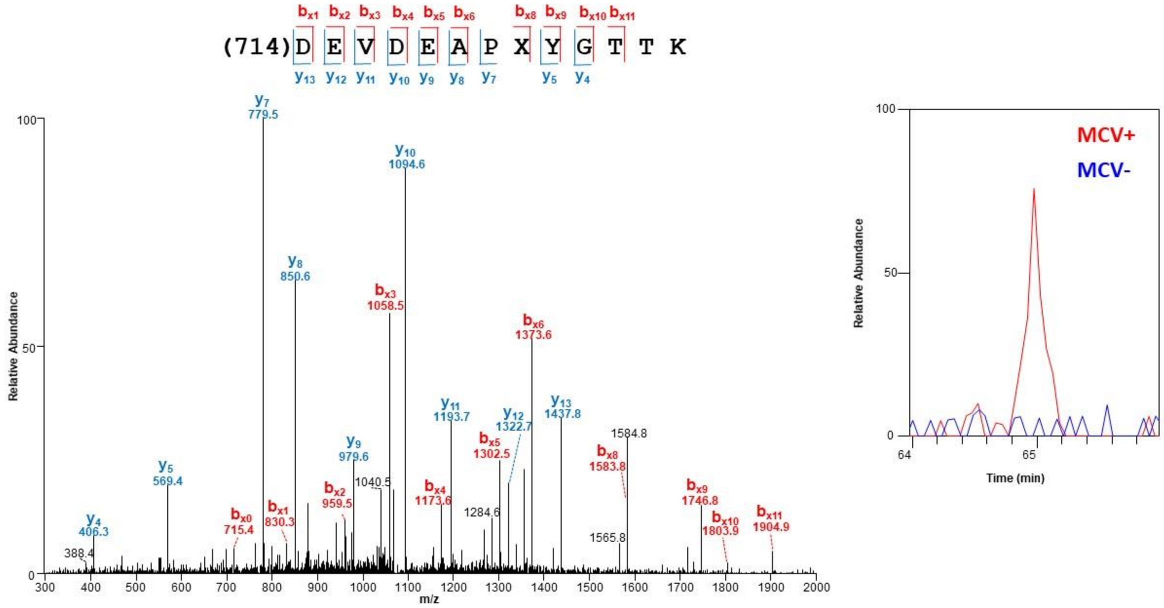

Tandem mass spectrum of proteomic feature #15 from Table 1. Spectrum was acquired by targeted collision activated dissociation (CAD). The tryptic peptide ion *m/z* 1076.5 (p-value 2.36E-05 and fold change 5405.8) has a manual *de novo* amino acid sequence tag (714)**DEVDEAPXYGTTK**. The amino acid “X” refer to isoleucine or leucine. Not enough information from the spectrum could determine the N-terminal amino acids. Representative MCV positive and negative shown as MS selected ion chromatogram.

Supplemental Figure 7

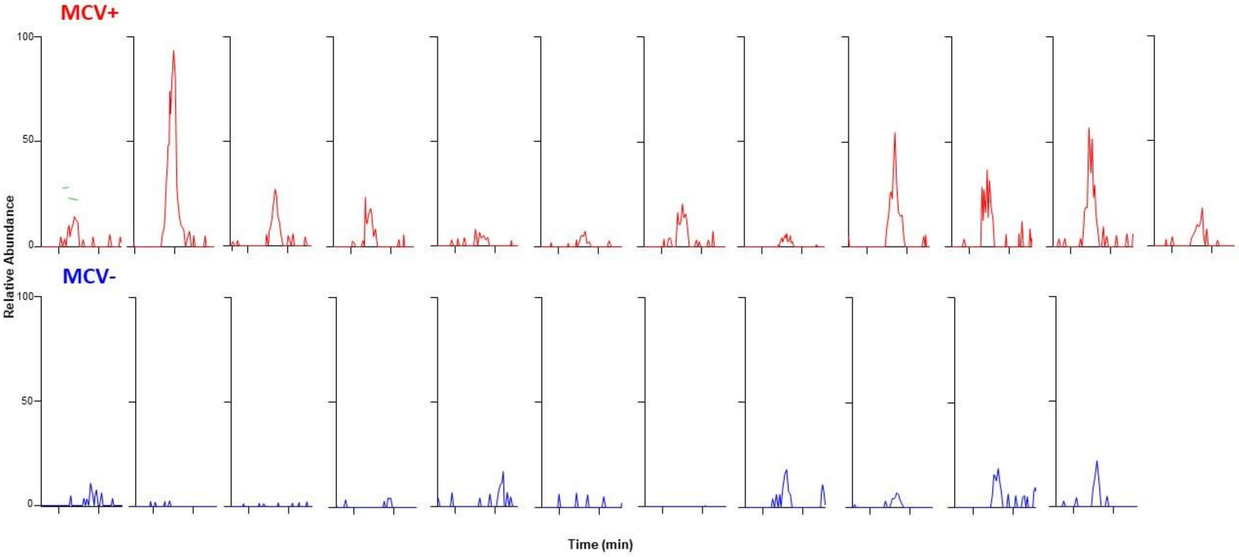

The abundance of peptide **AYEYGPNPH**(158)**NSR** (p-value 2.26E-19 and fold change 4316.9) in individual patient samples, shown as MS selected ion chromatogram from MCV positive and MCV negative groups. Each panel represents one individual FFPE sample.
